# Penttinen syndrome‐associated *PDGFRB* Val665Ala variant causes aberrant constitutive STAT1 signalling

**DOI:** 10.1111/jcmm.17427

**Published:** 2022-06-10

**Authors:** Audrey Nédélec, Emilie M. Guérit, Guillaume Dachy, Sandrine Lenglez, Lok San Wong, Florence A. Arts, Jean‐Baptiste Demoulin

**Affiliations:** ^1^ Experimental Medicine Unit, De Duve Institute Université catholique de Louvain Brussels Belgium

**Keywords:** ageing, congenital disease, imatinib, PDGFRB, Penttinen syndrome, platelet‐derived growth factor, STAT1, targeted therapy, tyrosine kinase inhibitors

## Abstract

Penttinen syndrome is a rare progeroid disorder caused by mutations in platelet‐derived growth factor (PDGF) receptor beta (encoded by the *PDGFRB* proto‐oncogene) and characterized by a prematurely aged appearance with lipoatrophy, skin lesions, thin hair and acro‐osteolysis. Activating mutations in *PDGFRB* have been associated with other human diseases, including Kosaki overgrowth syndrome, infantile myofibromatosis, fusiform aneurysms, acute lymphoblastic leukaemia and myeloproliferative neoplasms associated with eosinophilia. The goal of the present study was to characterize the *PDGFRB* p.Val665Ala variant associated with Penttinen syndrome at the molecular level. This substitution is located in a conserved loop of the receptor tyrosine kinase domain. We observed that the mutant receptor was expressed at a lower level but showed constitutive activity. In the absence of ligand, the mutant activated STAT1 and elicited an interferon‐like transcriptional response. Phosphorylation of STAT3, STAT5, AKT and phospholipase Cγ was weak or undetectable. It was devoid of oncogenic activity in two cell proliferation assays, contrasting with classical PDGF receptor oncogenic mutants. STAT1 activation was not sensitive to ruxolitinib and did not rely on interferon‐JAK2 signalling. Another tyrosine kinase inhibitor, imatinib, blocked signalling by the p.Val665Ala variant at a higher concentration compared with the wild‐type receptor. Importantly, this concentration remained in the therapeutic range. Dasatinib, nilotinib and ponatinib also inhibited the mutant receptor. In conclusion, the p.Val665Ala variant confers unique features to PDGF receptor β compared with other characterized gain‐of‐function mutants, which may in part explain the particular set of symptoms associated with Penttinen syndrome.

## 1 INTRODUCTION

Penttinen syndrome is a rare genetic disorder characterized by a prematurely aged appearance. The disease symptoms include skin atrophy with scars, lipoatrophy, thin hair, underdeveloped cheekbones, proptosis and marked acro‐osteolysis.^1^ Unlike other syndromes associated with ‘premature aging’, Penttinen syndrome does not result in premature death by cancer. The disease was linked to a de novo point mutation in *PDGFRB*, which encodes platelet‐derived growth factor (PDGF) receptor β, leading to a valine to alanine substitution at codon 665 (p.V665A).^1^, ^2^

*PDGFRB* is expressed at a particularly high level in mesenchymal stem cells, vascular smooth muscle cells, pericytes and fibroblasts. Ablation of *Pdgfrb* in mice results in vessel leakage, haemorrhages, kidney failure and perinatal lethality.^3^ This gene also plays a role in craniofacial development and bone regeneration.^4^, ^5^ *PDGFRB* is homologous to *PDGFRA*, which encodes PDGF receptor α. The latter binds to four dimeric ligands, namely PDGF‐AA, ‐AB, ‐BB and ‐CC, while PDGF receptor β binds to PDGF‐BB and ‐DD.^6^ Upon ligand binding, PDGF receptors dimerize and undergo a conformation change, which disrupts the inhibitory juxtamembrane domain (JMD) and activates the intracellular protein‐tyrosine kinase domain (TKD). This leads to phosphorylation of the receptor itself in addition to multiple signalling mediators.^6^ Major PDGF receptor signalling pathways include signal transducers and activators of transcription (STAT) factors, mitogen‐activated protein kinases, phospholipase Cγ (PLCγ), phosphatidylinositol‐3 kinase and AKT.^6^ Like many genes encoding receptor tyrosine kinases, *PDGFRA* and *PDGFRB* are proto‐oncogenes. Alterations in these genes have been described in myeloid malignancies associated with eosinophilia and B‐cell acute lymphoblastic leukaemia.^6^, ^7^ Somatic driver mutations in *PDGFRA* contribute to the development of glioma and gastrointestinal stromal tumours (GIST). Germline *PDGFRA* mutations cause an inherited disorder characterized by the combination of GIST and inflammatory fibroid polyps.^8^ In GIST and myeloid malignancies, tyrosine kinase inhibitors, such as imatinib, block PDGF receptors and were approved by regulatory agencies for the treatment of these neoplasms. *PDGFRB* mutations have also been identified in other neoplasms, including Castleman disease, mucinous histiocytosis and myofibroma.^9^, ^10^, ^11^, ^12^

Germline *PDGFRB* mutations are not restricted to Penttinen syndrome.^13^, ^14^ *PDGFRB* variants were associated with familial infantile myofibromatosis, characterized by the development of multiple myofibromas in children.^15^, ^16^ The most common variant in that disease is p.R561C, which weakly activates the receptor kinase activity and requires a second somatic hit for full receptor activation, the most frequent one being p.N666K.^9^ *PDGFRB* germline variants with a higher constitutive activity, such as p.P584R or p.W566R, have been identified in patients with Kosaki overgrowth syndrome, who exhibit alterations in bones, the brain and the skin.^17^, ^18^ More precisely, this syndrome features skeletal overgrowth, scoliosis, hyperelastic fragile skin, loss of subcutaneous adipose tissue, skull deformation, neuropsychiatric symptoms, arachnoid cysts, aneurysms and cerebral white matter lesions.^17^ Patients with Kosaki overgrowth syndrome often develop myofibromas, which are not observed in patients with Penttinen syndrome, although the p.V665A variant also confers constitutive signalling in the absence of ligand.^1^ The reason why this gain‐of‐function mutation induces such a unique phenotype remains unclear.

The goal of the present study was to compare mutant receptors to identify the molecular basis of the difference between these syndromes. We show that the *PDGFRB* p.V665A receptor, which we will refer to as βV665A, presents a deficient cell surface expression and signals preferentially via STAT1, inducing an interferon‐like molecular response.

## 2 MATERIAL AND METHODS

### 2.1 Cell culture and reagents

NIH3T3, HEK293T and γ2A cells were cultured in Dulbecco's modified Eagle medium (DMEM, ThermoFisher Scientific) supplemented with 10% foetal bovine serum (FBS) and 1% of penicillin–streptomycin solution (ThermoFisher Scientific). Ba/F3 cells were grown in DMEM supplemented with 10% FBS and IL‐3 (500 U/ml), produced by transfected CHO cells, as described.^19^ All cells grew at 37°C in a humidified atmosphere supplemented with 5% CO_2_. Antibodies are listed in Tables S1 and S2.

### 2.2 Mutagenesis, cell transfection and infection

The mutations p.P584R, p.R561C, p.W566R, p.V665A, p.N666H or p.N666K were introduced in human *PDGFRB* (RefSeq NM_002609.3) inserted into pcDNA3.1, pBabe‐puro or pEF/myc/cyto (Invitrogen) according to the QuickChange XL‐II kit protocol (Stratagene) using the oligonucleotides listed in Table S4. The nucleotide sequence of each construct was verified by Sanger sequencing (Eurofins).

Retroviral particles were produced in HEK293 BOSC cells cultured in Iscove's modified Dulbecco's medium (ThermoFisher Scientific) supplemented with 10% FBS. Three million cells were co‐transfected with 10 μg pCL‐Eco plasmid and 10 μg wild‐type or mutated *PDGFRB* cloned in pBABE‐puro by using TurboFect reagent (ThermoFisher Scientific). The supernatant containing viruses was harvested 48 h after transfection, filtrated and mixed with 10 μg/ml polybrene and added on two million NIH3T3 cells. After 24 h, cells were selected with puromycin (1 μg/ml, Sigma‐Aldrich) for 1 week.

Lentiviruses were produced in HEK293T cells. Three million cells were seeded in 10 cm plates. The day after, 18 μg of pLKO plasmid encoding a shRNA against STAT1 (Sigma, NM_009283) or scramble (Sigma, MFCD07785395) was co‐transfected with the packaging plasmids pCMV‐dr8.2 dvpr (10 μg), pCMV‐VSV‐G (6 μg) and pRSV‐Rev (6 μg) using the calcium phosphate precipitate method. Four hours later, cells were washed and incubated in culture medium for 48 h. Supernatants were harvested, filtrated and added on NIH3T3 cells with 10 μg/ml polybrene (Sigma). The shRNA efficacy was controlled by qPCR.

Ten million Ba/F3 cells were electroporated (200 V, 1300 μF, 75 Ohm) with 40 μg pEF/myc/cyto plasmid encoding wild‐type or mutant *PDGFRB*. The following day, electroporated cells were selected with G418 (3 mg/ml) for 7 days. Cell surface expression was tested by flow cytometry as described below. Cells were sorted to obtain stable cell lines expressing comparable levels of receptor.

### 2.3 Flow cytometry

NIH3T3 or Ba/F3 cells were harvested, washed in PBS and incubated with primary mouse anti‐human PDGFRB antibody (AH 17.2, 5 μg/ml) for 20 min at 4°C.^20^ Cells were then washed and incubated with secondary antibody coupled to phycoerythrin (#715–116‐150, Jackson Immunoresearch) for 20 min at 4°C in the dark. PDGFRB expression was analysed using a FACSVerse flow cytometer, and positive cells were sorted using FACSAria III (both from BD Biosciences).

### 2.4 Western blot experiments

HEK293T and γ2A cells were transiently transfected with wild‐type or mutant *PDGFRB* cloned in pEF/myc/cyto and with pMX‐mJAK2 (γ2A only), by using calcium phosphate (as described in^13^), Lipofectamine 2000 (ThermoFisher Scientific) or Fugene HD transfection reagent (Promega) according to manufacturers' instructions.

Cells were seeded in six‐well plates and starved for 7 h in medium without FBS before cell lysis. Cells were treated with human PDGF‐BB (25 ng/ml), IFNγ (25 ng/ml, both from PeproTech), imatinib (LC laboratories) or ruxolitinib (INCB18424, MedChemExpress) as indicated. After cell lysis, protein concentration was determined by using Pierce BCA protein assay kit (ThermoFisher). Identical amounts of proteins were diluted in Laemmli's sample buffer, boiled, separated in Novex precast gels (ThermoFisher Scientific) and blotted onto PVDF membranes using standard procedures. Membranes were first incubated with the antibody recognizing the phosphorylated form of the protein. Membranes were then striped in NaOH 0.4 M and re‐probed with an antibody targeting the corresponding protein. The list of antibodies is provided in Table S1. In experiments shown in Figures 3, 5 and 6, chemiluminescence was detected using Amersham Hyperfilm ECL (GE Healthcare). The other Western blot images were generated by a Fusion Solo 7S camera (Vilber).

For immunoprecipitation experiments, cell lysates were first incubated with primary anti‐PDGFRB antibody overnight at 4°C. The next day, Protein A/G UltraLink Resin (ThermoFisher Scientific) was added for 90 min at 4°C. Beads were washed twice in lysis buffer and once with PBS. Samples were resuspended in Laemmli's buffer and proteins were eluted from beads by boiling at 95°C for 5 min, followed by immunoblotting.

### 2.5 RNA extraction and quantitative RT‐PCR

Total RNA was extracted using Tripure reagent (Roche) according to the manufacturer's recommendations and treated with DNAse. Reverse transcription was performed with 1 μg of RNA using M‐MLV reverse transcriptase (28025013, ThermoFisher Scientific) and oligodT (Promega) in a 22 μl final reaction volume. Quantitative RT‐PCR was performed by using Absolute QPCR mix SYBR Green, with fluorescein (AB1220A, ThermoFisher Scientific) with 100 nM of primers (see Table S3), designed by using Primer 3 software. Reactions were carried out in triplicate in a 96‐well plate, using the CFX96 Touch Real‐Time PCR detection system (Bio‐Rad). Results were quantified using the ΔΔCT method and normalized against actin expression levels.

### 2.6 Foci formation assay

NIH3T3 cells were transfected with 0.55 μg wild‐type or mutant *PDGFRB* cloned in pcDNA.3.1 with Lipofectamine 2000 according to the manufacturer's instructions. Cells were selected with G418 and processed as previously described.^21^ Three weeks after transfection, foci were fixed in methanol and stained with 0.2% of crystal violet in 20% ethanol. Foci density was quantified with Quantity One software (Bio‐Rad).

### 2.7 Thymidine incorporation assay

Ba/F3 cells stably expressing wild‐type or mutant *PDGFRB* were washed three times with DMEM without growth factors. Cells were seeded at 20,000 cells/well in 96‐well plates (seven replicates per condition). PDGF‐BB, IL‐3 or imatinib was added as indicated. After 20 h, 0.5 μCi [^3^H]‐thymidine (Perkin Elmer) was added in each well and incubated at 37°C for 4 h. Thymidine incorporation was quantified using a TopCount instrument (Perkin Elmer).

### 2.8 Luciferase reporter assays

γ2A and HEK293T cells were seeded in 48‐well plate (5 × 10^5^ cells/well). Cells were co‐transfected by Lipofectamine 2000 according to the manufacturer's procedure with wild‐type or mutant *PDGFRB*, a GRR5 or SRE luciferase reporter, and a pRLT‐TK renilla luciferase reporter (Promega) in triplicates. In addition, pMX‐mouse JAK2 and pMX‐mouse STAT5 were co‐transfected in γ2A.^22^ Eight hours after transfection, cells were cultured in medium supplemented with FBS and PDGF‐BB (25–50 ng/ml). After 24 h, cells were lysed in lysis buffer (dual‐luciferase assays, Promega). The firefly luciferase activity was divided by renilla luciferase activity. Both were measured using a GLOMAX instrument (Turner Biosystems, Promega). The average luciferase ratio was normalized to the wild‐type condition.

### 2.9 Statistics

All experiments were repeated at least three times with comparable results. Statistical analyses to compare two specific conditions in a given assay were performed using a Student's *t*‐test in GraphPad Prism version 9.0.1, unless stated otherwise. The average of multiple independent experiments is shown with standard error of the mean (SEM) (*p*‐values: *p < 0.05; **p < 0.01; ***p < 0.001; ****p < 0.0001; ns, non‐significant).

## 3 RESULTS

### 3.1 The p.V665A receptor mutant is constitutively active but not oncogenic

**FIGURE 1:**
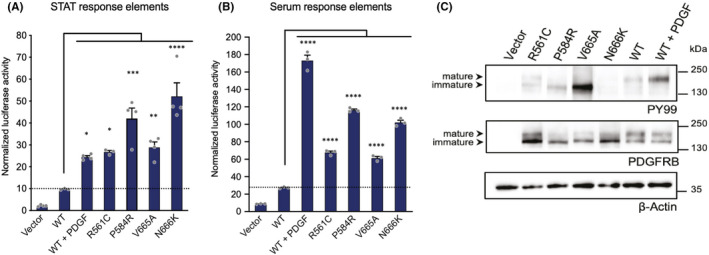
*PDGFRB* p.V665A mutant is constitutively active. The activity of the mutants was analysed in a dual‐luciferase reporter assay. γ2A (A) or HEK293T (B) cells were co‐transfected with wild‐type (WT) or mutant *PDGFRB* and a firefly luciferase reporter controlled by STAT‐response elements (A) or serum‐response elements (B), respectively. Results were normalized using a renilla luciferase control reporter. As positive control, cells expressing the WT receptor were stimulated with PDGF‐BB. The mean of four independent experiments is shown with SEM. Statistical analysis was performed using an anova one‐way test with Dunnett correction to compare mutant to wild‐type conditions. (C) HEK293T cells were transfected with WT or mutant *PDGFRB* or the empty vector and starved for 7 h before lysis. As positive control, cells expressing the WT receptor were stimulated with PDGF‐BB for 15 min. Total cell lysates were analysed by Western blotting. The receptor phosphorylation was probed with an anti‐phospho‐tyrosine antibody. Membranes were re‐probed with anti‐PDGFRB and anti‐Actin antibodies. Representative blots are shown (*n* = 4)

We first compared the activity of βV665A with that of other variants (presented in Table 1), in a luciferase reporter assay, as a readout of STAT transcription factor activation. Figure 1A shows that βV665A was active in the absence of ligand. We obtained a similar result using a luciferase reporter assay sensitive to serum response factor (Figure 1B). In this second assay, the activity of βV665A was similar to βR561C, but lower than βP584R, βN666K or the stimulated wild‐type receptor. Western blot experiments showed that transfected HEK293T cells expressed the variants, with a decrease in mature fully glycosylated receptor (upper band) compared with wild‐type receptor (Figure 1C), in agreement with a previous report.^1^ Blotting with an anti‐phosphotyrosine antibody showed that the βV665A mutant was phosphorylated in the absence of stimulation. Collectively, these experiments confirmed that the p.V665A mutation conferred a gain of receptor function.

**TABLE 1:** *PDGFRB* mutations included in the study

Mutation	Disease	Type	Domain	Oncogenic	References
p.R561C	Infantile myofibromatosis	Germline familial	JMD	Yes	[15, 16]
p.W566R	Kosaki syndrome & myofibroma	Germline, de novo	JMD	Yes	[37]
p.P584R	Kosaki syndrome & myofibroma	Germline, de novo	JMD	Yes	[18]
p.V665A	Penttinen syndrome	Germline, de novo	TKD	No	[36]
p.N666K	Myofibroma	Somatic	TKD	Yes	[9]
p.N666H	Mixed syndrome	Germline, de novo	TKD	Yes	[35]

Abbreviations: DMEM, Dulbecco's modified Eagle medium; FBS, foetal bovine serum; GIST, gastrointestinal stromal tumour; IFN, interferon; JMD, juxtamembrane domain; PDGF, platelet‐derived growth factor; PLCγ, phospholipase C γ; STAT, signal transducer and activator of transcription; TKD, tyrosine kinase domain; TKI, tyrosine kinase inhibitor.

**FIGURE 2:**
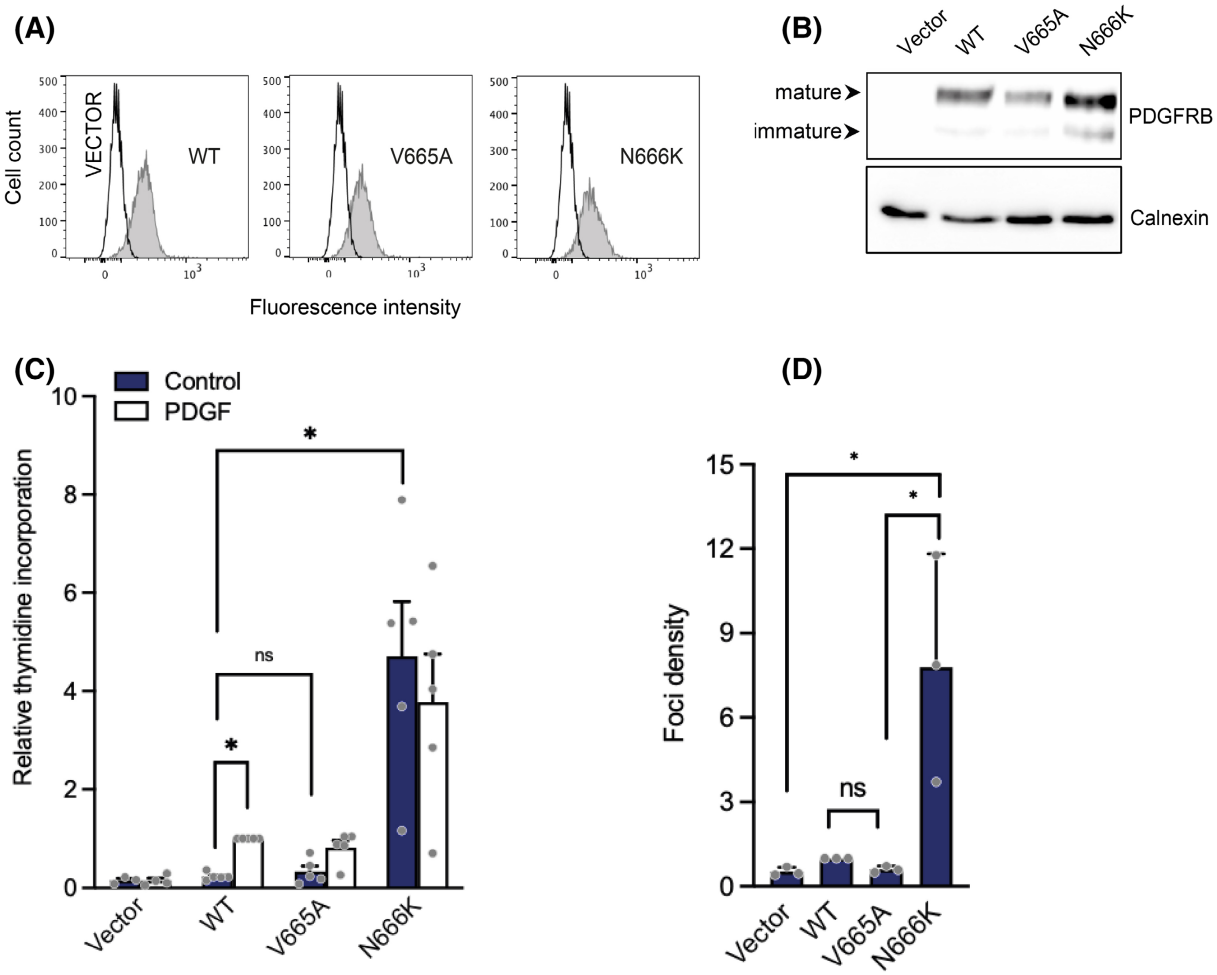
p.V665A mutant receptor does not stimulate cell proliferation. (A) Ba/F3 cells were electroporated with *PDGFRB* (WT, V665A or N666K) or an empty vector. Cells were selected in the presence of G418 and sorted. The receptor cell surface expression was assessed by flow cytometry with a human PDGFRB‐specific antibody. (B) Transfected Ba/F3 cell lysates were analysed by Western blotting using anti‐PDGFRB antibodies. Membranes were re‐probed with an anti‐calnexin antibody as loading control. (C) Transfected Ba/F3 cells were stimulated with control medium, PDGF‐BB or IL‐3. After 20 h, [^3^H]‐thymidine was added to each well for 4 h. Incorporation of radiolabelled thymidine into cell DNA was quantified. Results were normalized using the stimulated wild‐type condition as reference. The average of five independent experiments is shown with SEM. All cell lines responded similarly to IL‐3 (not shown). (D) NIH3T3 cells were transfected in triplicates with *PDGFRB* (WT, V665A or N666K). Three weeks after transfection, foci were stained with crystal violet and quantified. The mean of three independent experiments is shown with SEM

We had previously demonstrated that βR561C, βP584R and βN666K, like all characterized *PDGFRB* activating mutations, had an oncogenic activity in Ba/F3 and NIH3T3 cells, consistent with a role in myofibroma development.^23^ To our knowledge, the ability of βV665A to stimulate proliferation has not been explored. We first stably transfected Ba/F3 cells and sorted them to obtain cell lines expressing similar receptor levels (Figure 2A‐B). Surprisingly, βV665A expression did not support Ba/F3 cell proliferation in the absence of growth factor, in contrast to βN666K, used as positive control (Figure 2C). We have shown that transfection of oncogenic *PDGFRB* mutants, such as βR561C, βP584R or βN666K, resulted in long‐term interleukin‐3‐independent growth of Ba/F3 cells.^23^ This was not observed in cells expressing βV665A, which died in the absence of growth factor (data not shown). In line with this observation, transient βV665A transfection in NIH3T3 did not promote foci formation, in another classical cell transformation assay (Figure 2D). Taken together, our results indicated that βV665A was a unique gain‐of‐function mutant, devoid of oncogenic activity.

### 3.2 βV665A preferentially activates STAT1

**FIGURE 3:**
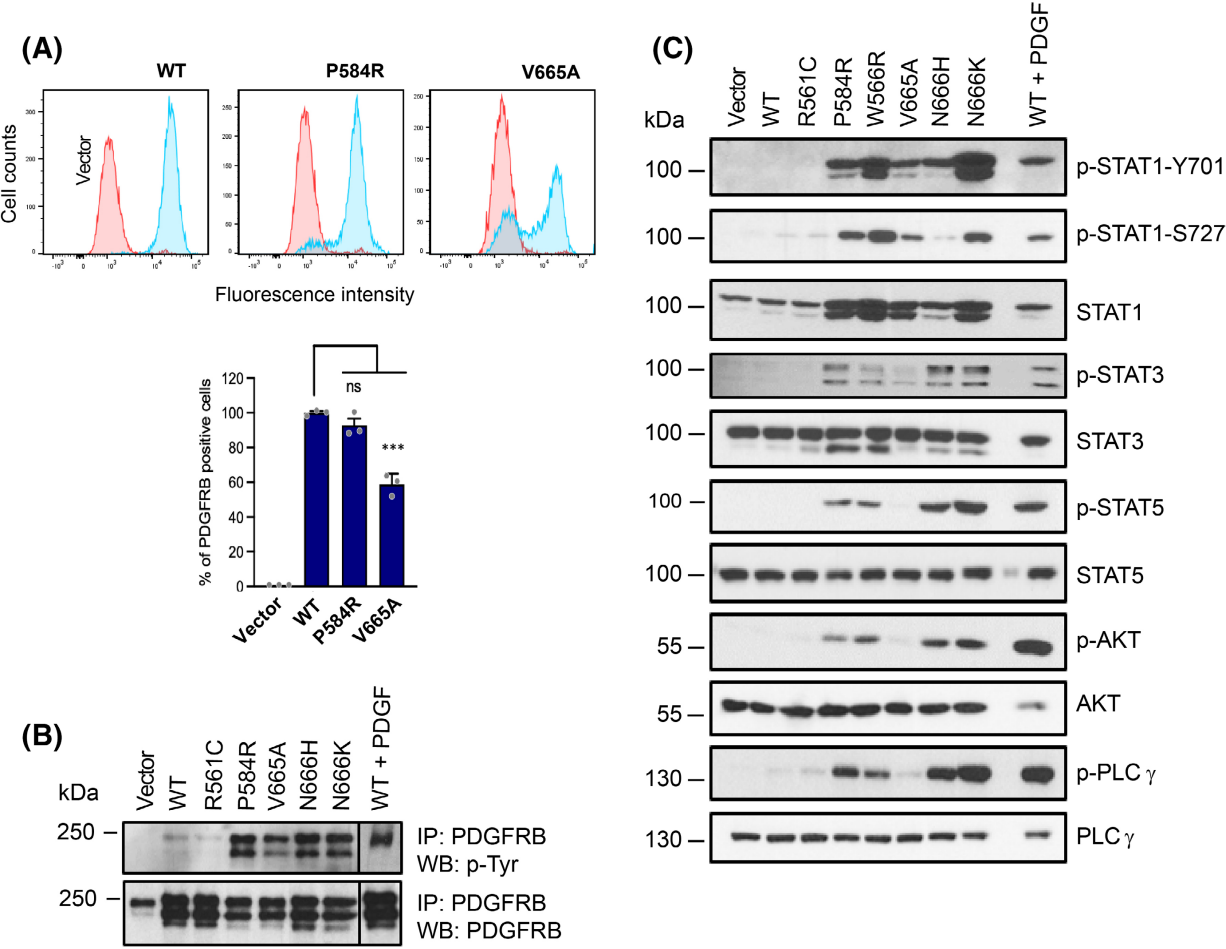
p.V665A mutant preferentially activates STAT1. (A) Human PDGF receptor β expression in transduced NIH3T3 cells was tested by flow cytometry (blue) and compared to cells transduced with the empty vector (red). The average percentage of positive cells was calculated from three experiments with SEM. The receptor expression levels of the other cell lines used in the study are shown in Figure S1. (B) PDGF receptor β phosphorylation and expression levels. Transduced NIH3T3 cells were starved for 7 h. As a positive control, cells expressing WT receptors were stimulated with PDGF‐BB (25 ng/ml) for 15 min before lysis. The receptor was isolated by immunoprecipitation and analysed by Western blot using anti‐phospho‐tyrosine and anti‐PDGFRB antibodies. One representative blot out of three is shown. (C) NIH3T3 cells were treated as in (B). Total cell lysates were analysed by Western blot with the indicated antibodies (*n* = 3)

We next transduced NIH3T3 fibroblasts with different receptor mutants to obtain stable cell lines. We controlled their expression levels by flow cytometry (Figure 3A). This procedure usually produces homogenous cell populations. However, a lower percentage of βV665A‐expressing cells was consistently observed, in multiple independent experiments (Figures 3A and S1). As expected, the mutant receptors were constitutively phosphorylated on tyrosines (Figure 3B). We next analysed the phosphorylation of various PDGF receptor signalling mediators, including STAT transcription factors, AKT and PLCγ. Figure 3C shows that βV665A, like other variants, induced a strong phosphorylation of STAT1. As expected, the total amount of STAT1 protein was also increased, since sustained STAT1 activation reportedly enhances *STAT1* gene transcription.^9^, ^24^ By contrast, phosphorylation of STAT3 and PLCγ was weaker in cells expressing βV665A compared with other gain‐of‐function mutants or with cells stimulated with PDGF‐BB. Phosphorylation of STAT5 and AKT was not detectable in NIH3T3‐βV665A cells. Similar results were obtained with cells that were sorted to express the same level of βV665A and wild‐type receptor (data not shown).

### 3.3 βV665A elicits an interferon‐like transcriptional response

**FIGURE 4:**
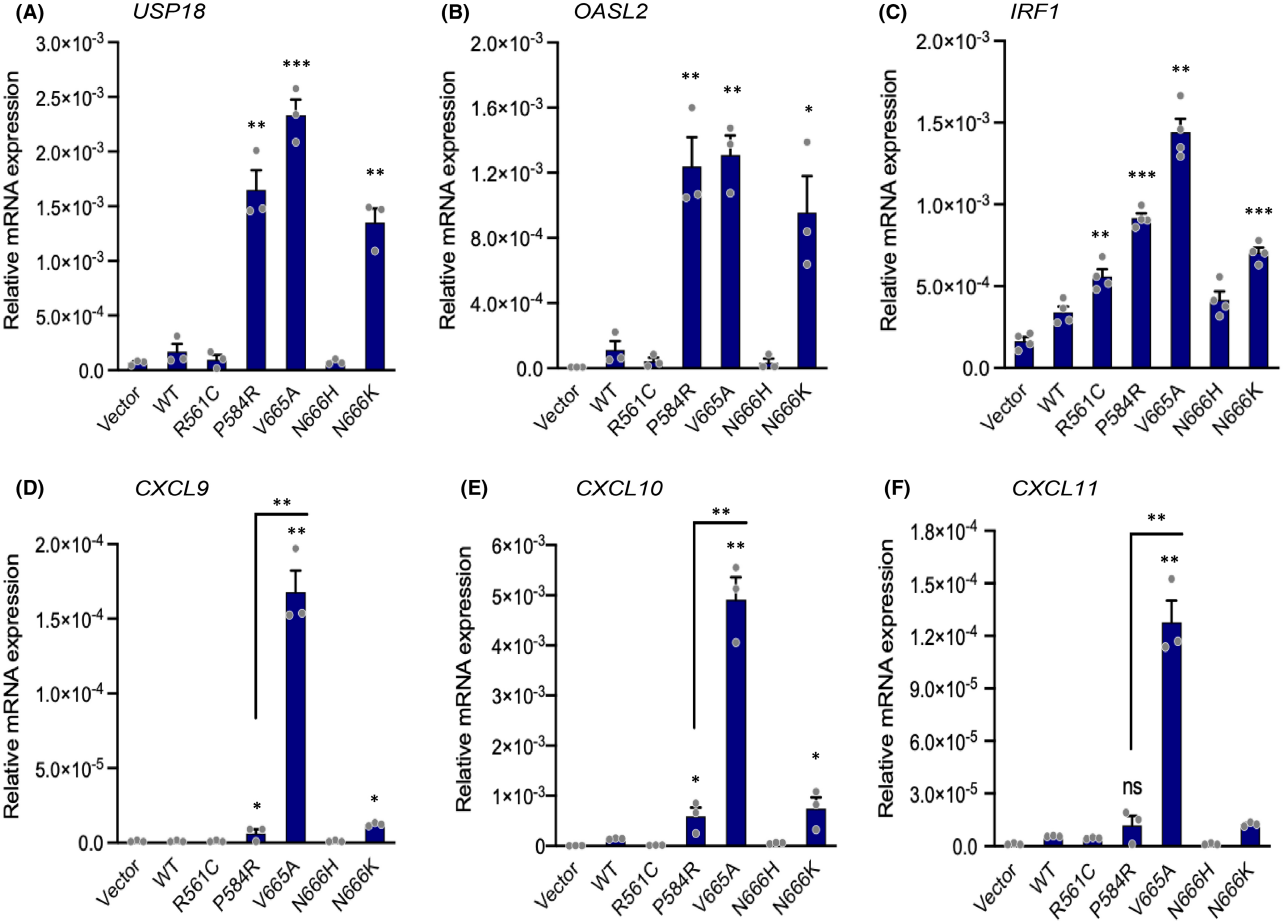
Regulation of interferon target genes by βV665A. NIH3T3 cells expressing the indicated receptor were produced as described in Figure 3. Cells were cultured in serum‐free medium for 12 h before RNA extraction. The expression of each target gene was assessed by RT‐qPCR. Expression values were normalized with those obtained for murine β‐Actin. The mean of three independent experiments (arbitrary units) is shown with SEM. Statistical analysis was performed using Student *t*‐test to compare each mutant to the wild‐type condition

**FIGURE 5:**
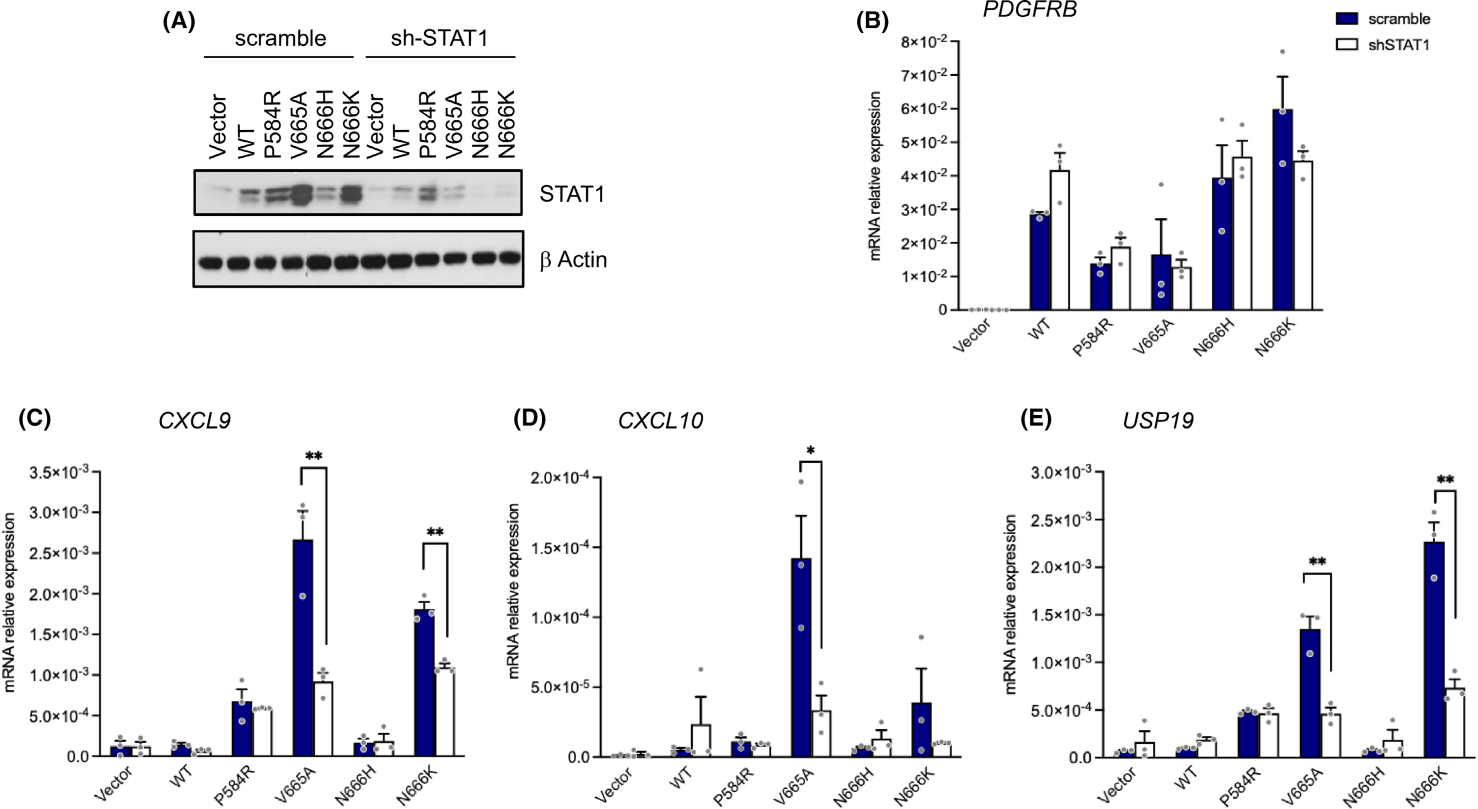
STAT1 is required for interferon target gene regulation by βV665A. NIH3T3 cell lines expressing the indicated receptor were produced as shown in Figure 3. Cells were infected with a lentiviral vector to express shRNA against STAT1 (white bars) or a control shRNA (scramble, dark blue bars). (A) The expression of STAT1, PDGFRB and β‐Actin was tested by Western blotting. Expression of human *PDGFRB* (B) and interferon target genes (C–E) was determined by quantitative RT‐qPCR. Results are expressed as relative mRNA expression normalized to mouse β‐Actin. The mean of three independent experiments (arbitrary units) is shown with SEM

To investigate further the role of STAT1 downstream βV665A, we compared the transcriptome of fibroblasts expressing βV665A, βP584R, βR561C or the wild‐type receptor. Gene expression analysis revealed a strong interferon response triggered by βV665A and βP584R (Figure S2). These results were confirmed by measuring the expression of classical interferon target genes, namely IRF1, USP18 and OASL2 (Figure 4). Interestingly, βV665A more specifically increased the mRNA levels of three chemokines, CXCL9, CXCL10 and CXCL11. We next determined whether STAT1 mediated the regulation of these genes by using specific inhibitory shRNA, which strongly reduced STAT1 protein expression (Figure 5A). STAT1 knockdown blunted the upregulation of CXCL9, CXCL10 and UPS18 in cells expressing βV665A (Figure 5C–E). As expected, the expression level of *PDGFRB* was not affected by these shRNA (Figure 5B).

**FIGURE 6:**
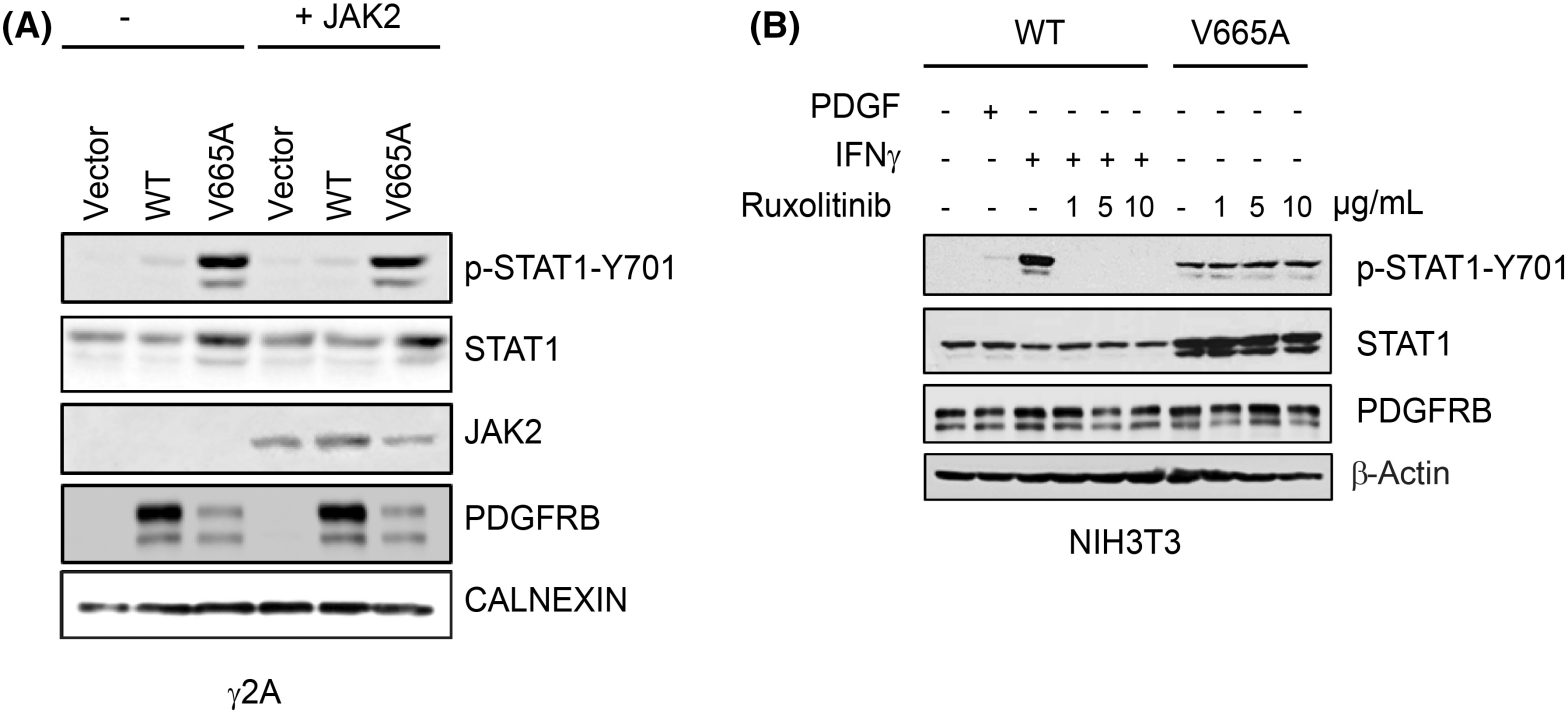
STAT1 activation by βV665A is JAK2‐independent. (A) γ2A cells were transiently transfected with *PDGFRB* (WT or V665A) and JAK2, as indicated. After transfection, cells were starved for 7 h and lysed. The total lysates were analysed by Western blotting. (B) NIH3T3 cell lines expressing the indicated receptor were produced as shown in Figure 3. Cells were starved for 7 h and treated with different concentrations of ruxolitinib as indicated. As positive controls, cells expressing the WT receptor were stimulated with PDGF‐BB or IFNγ for 15 min. The total lysates were analysed by Western blotting

We next investigated the mechanism whereby βV665A triggered this interferon transcriptional response. The activation of STAT1 by interferons critically depends on JAK kinases. Several reports indicate that PDGF can activate JAK2, which may contribute to STAT activation in some cell types.^6^ To test the possibility that βV665A activates STAT1 via JAK2, we first used γ2A fibrosarcoma cells, which lack JAK2 and are therefore unresponsive to interferon γ. In line with the results obtained in NIH3T3 cells, the expression of mature fully glycosylated βV665A was reduced in these cells compare with wild‐type receptor (Figure 6A). We observed a strong phosphorylation of STAT1 upon γ2A transfection with βV665A, which was not modified by restoring JAK2 expression. In a parallel set of experiments, we used the JAK1/JAK2 inhibitor ruxolitinib, which is a potent inhibitor of interferon signalling. As expected, ruxolitinib blocked STAT1 phosphorylation in fibroblasts treated with interferon γ (Figure 6B). By contrast, JAK inhibition had no impact on STAT1 phosphorylation in cells expressing βV665A. Our results demonstrated that STAT1 activation by this mutant receptor was independent of JAK2 and interferon receptor signalling.

### 3.4 The p.V665A variant decreases the receptor sensitivity to imatinib

**FIGURE 7:**
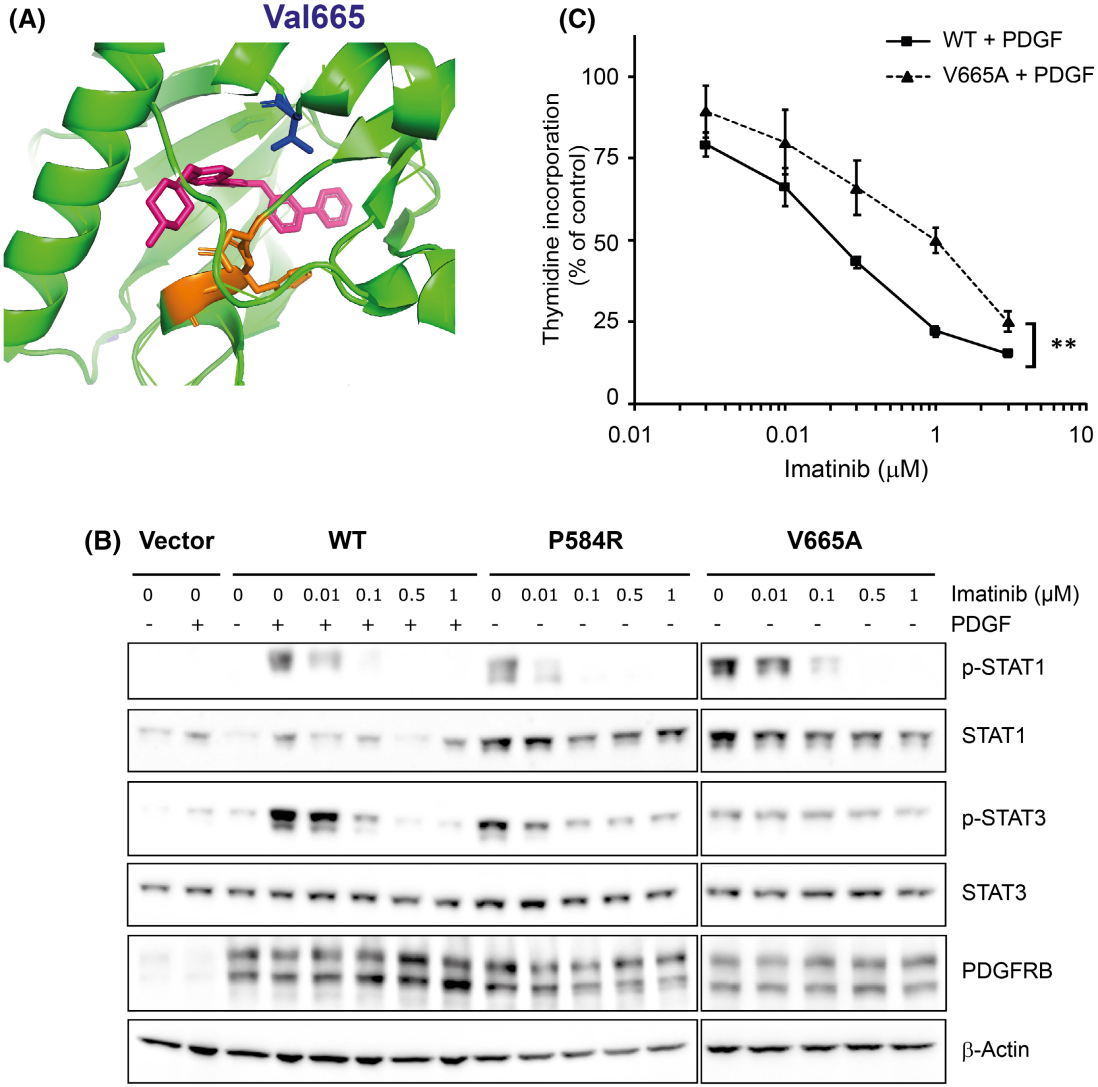
p.V665A mutation decreases the sensitivity to imatinib. (A) Position equivalent to valine 665 (blue), in the catalytic cleft of the PDGFRA kinase domain (green) co‐crystalized with imatinib (pink). The Asp‐Phe‐Gly (DFG) motif, which plays an important role in the regulation of kinase activity, is shown in orange. The alpha‐C helix is on the left. Drawn using PyMol based on structure #6JOL from Protein Data Bank. (B) NIH3T3 cell lines expressing the indicated receptor were produced as shown in Figure 3. Cells were starved for 4 h and treated with different concentrations of imatinib as indicated. As a positive control, cells expressing the WT receptor were stimulated with PDGF‐BB for 15 min. The total lysates were analysed by Western blotting. (C) Ba/F3 cells expressing WT PDGFRB or βV665A were produced as described in Figure 2. Cells were stimulated with PDGF‐BB for 24 h in the presence of the indicated concentration of imatinib. Four hours before harvest, [^3^H]‐thymidine was added to each well. Radiolabelled thymidine incorporation was quantified and compared to cells that were not treated with imatinib. The average of three independent experiments is shown with SEM and statistical analysis (anova test, *p* < 0.01)

Valine 665 is located close to the ATP‐binding pocket of the PDGF receptor β kinase domain and may thereby affect the sensitivity to tyrosine kinase inhibitors, such as the ATP competitor imatinib (Figure 7A). Since this molecule has been approved for the treatment of several diseases driven by aberrant PDGF receptor signalling,^6^ it may represent a valuable therapeutic option for severe Penttinen syndrome. It was therefore important to test the impact of the p.V665A substitution on cell responsiveness to imatinib. When used at a clinically relevant concentration of 1 μM on NIH3T3 cells, imatinib efficiently blocked STAT1 phosphorylation downstream βV665A and βP584R (Figure 7B). The βP584R and wild‐type receptors were more sensitive to imatinib compared with βV665A. To investigate further this difference, we compared the effect of imatinib on the proliferation of Ba/F3 cells stimulated by PDGF and expressing either the wild‐type receptor or βV665A. Data shown in Figure 7C confirmed that higher imatinib concentrations were required to block βV665A, compared with the wild‐type receptor. Finally, we tested several other tyrosine kinase inhibitors that are currently approved for the treatment of adult chronic myeloid leukaemia, namely dasatinib, nilotinib and ponatinib. All of them significantly blocked the βV665A activity (Figure S3). Collectively, these results show that several approved tyrosine kinase inhibitors could target βV665A.

## 4 DISCUSSION

Our results demonstrate that the p.V665A substitution confers unique properties to the PDGF receptor β. First, we confirmed that p.V665A is a gain‐of‐function mutant. Several groups had reported that p.V665A confers constitutive signalling in the absence of ligand, illustrated by the phosphorylation of the receptor itself, STAT1, STAT3 and PLCγ.^1^, ^25^ By comparing this variant with other mutants in the same experiments, we confirmed that βV665A strongly activated STAT1. However, phosphorylation of other signalling mediators was much reduced (STAT3 and PLCγ) or absent (STAT5 and AKT) in cells expressing βV665A compared with other mutant receptors. The decreased ability to activate these pathways was not due to lower PDGFRB levels since it was also observed in sorted NIH3T3 cells with equivalent receptor expression.

We also observed that the p.V665A variant regulated some STAT1 target genes, such as the CXCL9/10/11 chemokines, to a higher extent. This could not be explained by differences in the intensity of STAT1 phosphorylation. Ho and Ivashkiv showed that STAT3 activation limits the upregulation of IRF1, CXCL9 and CXCL10 by interferon, by forming STAT1:STAT3 heterodimers.^26^ This observation suggests that the sustained activation of STAT3 by oncogenic *PDGFRB* mutants may dampen the interferon response.

The mechanism of STAT1 activation by βV665A did not rely on the activation of interferon γ signalling, since JAK2 was dispensable for STAT1 phosphorylation and gene regulation by βV665A. Although in vivo experiments have not revealed any role for STAT1 in PDGF physiological functions, several reports showed that PDGF receptor β can transiently activate this factor.^6^ In a cell‐free system, Vignais and Gilman^27^ showed that PDGF receptors could directly phosphorylate STAT1. We speculate that the p.V665A mutation may change the substrate specificity of the PDGF receptor β kinase domain, as observed previously for other receptor tyrosine kinases.^28^ This could modify the range of tyrosines that are phosphorylated in the receptor itself, in adaptor proteins and in signalling mediators. Alternatively, βV665A may signal from a different subcellular compartment or activate a negative regulator of PDGF signalling, for instance a phosphatase. Further studies are needed to decipher the exact mechanism of βV665A signalling.

Importantly, STAT1 activation may explain some of the features of Penttinen syndrome, as suggested by a recent report by Olson and colleagues.^25^ They introduced a *Pdgfrb* gain‐of‐function D849V mutation in mice, which triggered lethal inflammation. Deletion of one *Stat1* allele resulted in a tissue wasting phenotype, reminiscent of Penttinen syndrome. Mice with a homozygous *Stat1* deletion develop progressive overgrowth. These results suggested that the intensity of STAT1 signalling may determine the phenotypic outcomes. Our observations complement these findings by showing that the human p.V665A variant indeed favours STAT1 signalling compared with other *PDGFRB* germline mutations.

By contrast to most other *PDGFRB* gain‐of‐function mutants,^9^, ^10^, ^14^, ^23^, ^29^ βV665A did not transform Ba/F3 or NIH3T3 cells. This unique feature may stem from biased signalling. Indeed, unlike *STAT3* and *STAT5*, *STAT1* is not considered as a proto‐oncogene. STAT1 signalling even has anti‐tumour activity in different models.^30^ In addition, βV665A poorly activated AKT and PCLγ, which are classical oncogenic pathways. The lack of βV665A oncogenic activity fits with the reported absence of tumour development in patients with Penttinen syndrome.^1^ In contrast, about half of the patients with Kosaki overgrowth syndrome develop myofibromas.^18^, ^31^

In addition to biased signalling, we consistently observed a reduced expression of cell surface mature p.V665A receptor in NIH3T3 and γ2A cells, in agreement with the results of Johnston and colleagues,^1^ obtained in transiently transfected HeLa cells. Proteasome and lysosome inhibitors did not restore the receptor expression (data not shown), suggesting that increased degradation was not involved. Instead, constitutive activation of the kinase domain in the secretory pathway may perturb the receptor traffic to the cell surface, as previously reported for PDGF receptor α and other type‐III receptor tyrosine kinases.^7^, ^32^ We hypothesize that aberrant phosphorylation of endoplasmic reticulum or Golgi proteins may delay the receptor progression to the plasma membrane.

Penttinen syndrome is a severe debilitating condition.^1^, ^2^ The identification of causal *PDGFRB* mutations opened the possibility of treating this disease with tyrosine kinase inhibitors. However, point mutations in the kinase domain may strongly affect the sensitivity to such inhibitors. Here, we show that βV665A remained sensitive to imatinib at clinically relevant concentrations. Nevertheless, we also observed a reduced sensitivity to the drug compared with wild‐type receptor. We had previously demonstrated that the p.N666K mutation (the next residue in the PDGF receptor β polypeptide) also required higher imatinib concentration. In addition to imatinib, we showed that dasatinib, nilotinib and ponatinib also block βV665A activity. Dasatinib and nilotinib have been recently approved for the treatment of childhood leukaemia.^33^ Recent case reports suggest that imatinib and dasatinib may provide clinical benefits in two patients with Penttinen syndrome.^31^, ^34^ In the second one, dasatinib elicited a better response than imatinib. Future studies on a larger number of patients are required to draw definitive conclusions on the use of TKI in Penttinen syndrome. Possible long‐term side effects of these drugs in children include growth retardation.^33^, ^35^ Although ruxolitinib was recently approved for several inflammatory diseases, our results do not support the use of this TKI in Penttinen syndrome.

Beside p.V665A, other variants were identified in Penttinen‐like conditions. We described a case with a germline p.N666H variant and combined features of Penttinen syndrome, Kosaki syndrome and myofibromatosis.^35^ Another patient was reported with a severe form of Penttinen syndrome and a p.N666S *PDGFRB* variant.^36^ We also identified this variant in a case of myofibromatosis.^10^ Whether the phenotypic differences between these unique cases result from different properties of the variants, or from other individual variations remains to be determined.

Collectively, our results demonstrate that the p.V665A variant produces a unique set of perturbations at the molecular level, including biased STAT1 signalling, lack of oncogenic activity and decreased cell surface expression. In conclusion, p.V665A is a neomorphic variant, rather than a classical gain‐of‐function mutant, which can be targeted by TKI.

## AUTHOR CONTRIBUTIONS

**Audrey Nédélec:** Data curation (equal); formal analysis (lead); investigation (equal); methodology (equal); writing – review and editing (equal). **Emilie M. Guérit:** Formal analysis (equal); investigation (equal); writing – review and editing (equal). **Guillaume Dachy:** Formal analysis (equal); investigation (equal); visualization (equal). **Sandrine Lenglez:** Formal analysis (equal); investigation (equal); project administration (equal); writing – review and editing (equal). **Lok San Wong:** Formal analysis (equal); investigation (equal). **Florence A. Arts:** Formal analysis (equal); investigation (equal). **Jean‐Baptiste Demoulin:** Conceptualization (equal); funding acquisition (lead); methodology (equal); project administration (lead); supervision (equal); visualization (equal); writing – original draft (lead).

## CONFLICT OF INTEREST

The authors did not declare conflicts of interest.

## Supporting information


Figure S1

Figure S2

Figure S3

Tables S1‐S4

## Data Availability

The data that support the findings of this study are available from the corresponding author upon reasonable request.
