# Supplementary material for: Penttinen syndrome‐associated PDGFRB Val665Ala variant causes aberrant constitutive STAT1 signalling
Source: J Cell Mol Med. 2022 Jun 10;26(14):3902–12. doi: 10.1111/jcmm.17427 (PMC9279580; doi:10.1111/jcmm.17427)
Supplement: Supplementary file 1 — Figure S1 [file JCMM-26-3902-s004.pdf]

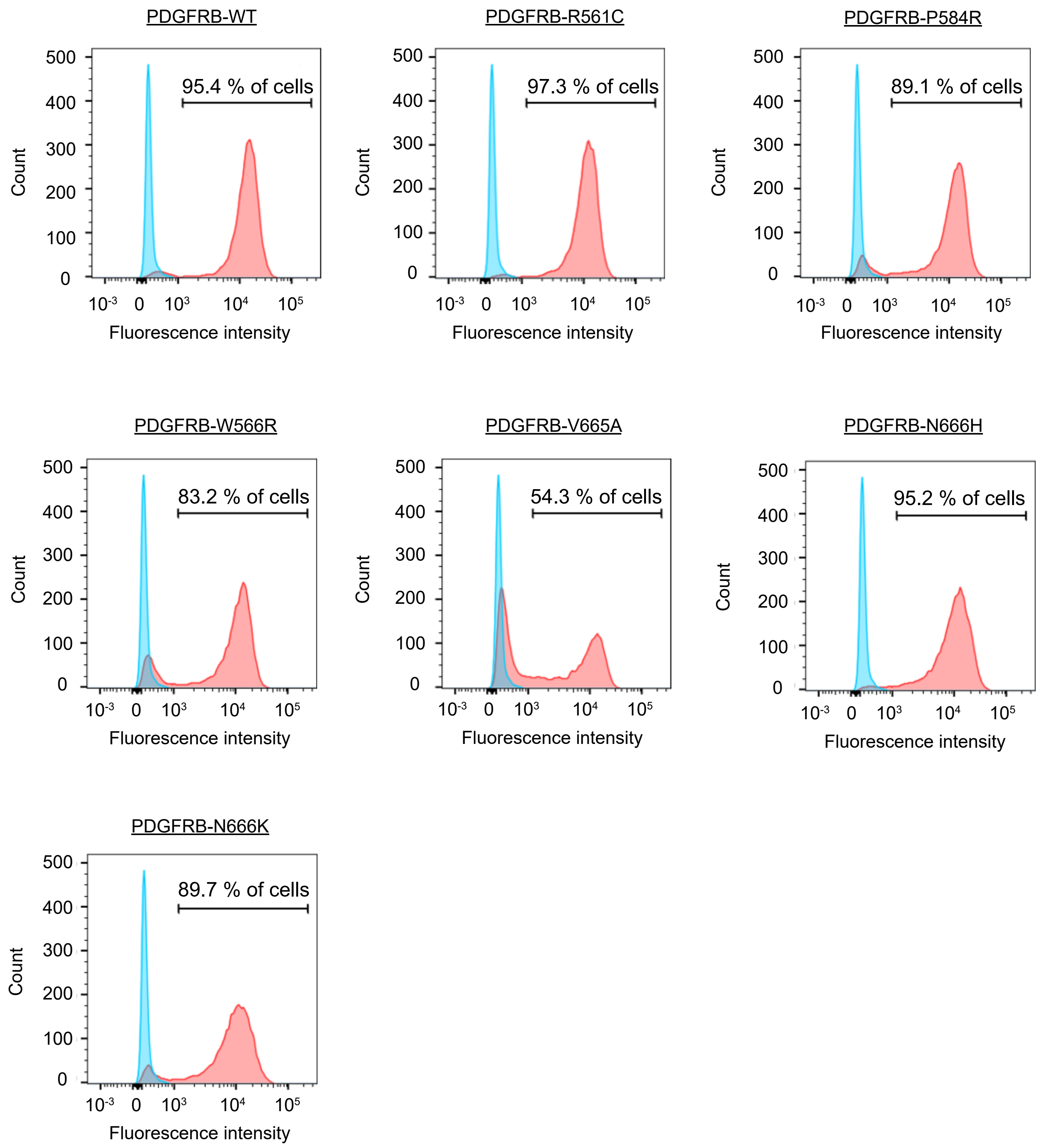

Supplementary Figure S1.
Cell surface expression of WT and mutant PDGFRB in NIH3T3 cells.

NIH3T3 cells were transduced with retroviral particles encoding PDGFRB WT, R561C, P584R, W566R, V665A, N666H or N666K . Cells were selected in the presence of puromycin for 10 days and analyzed by flow cytometry. The expression level of the receptors was detected using an anti-PDGFRB antibody as described in the method section. One representative experiment is shown.
