# Supplementary material for: Penttinen syndrome‐associated PDGFRB Val665Ala variant causes aberrant constitutive STAT1 signalling
Source: J Cell Mol Med. 2022 Jun 10;26(14):3902–12. doi: 10.1111/jcmm.17427 (PMC9279580; doi:10.1111/jcmm.17427)
Supplement: Supplementary file 2 — Figure S2 [file JCMM-26-3902-s001.pdf]

Supplementary figure S2

A

Term	Count	%	P-Value
cellular response to interferon-beta	23	18	8,9E-41
defense response to virus	25	19,5	2,2E-28
immune system process	27	21,1	3,6E-22
GTPase activity	23	18	5,4E-22
response to virus	17	13,3	3,5E-21
GTP binding	27	21,1	4,9E-21
innate immune response	26	20,3	1,9E-20
defense response	14	10,9	3,1E-14
defense response to protozoan	9	7	2,1E-12
cellular response to interferon-gamma	10	7,8	7,2E-11

B

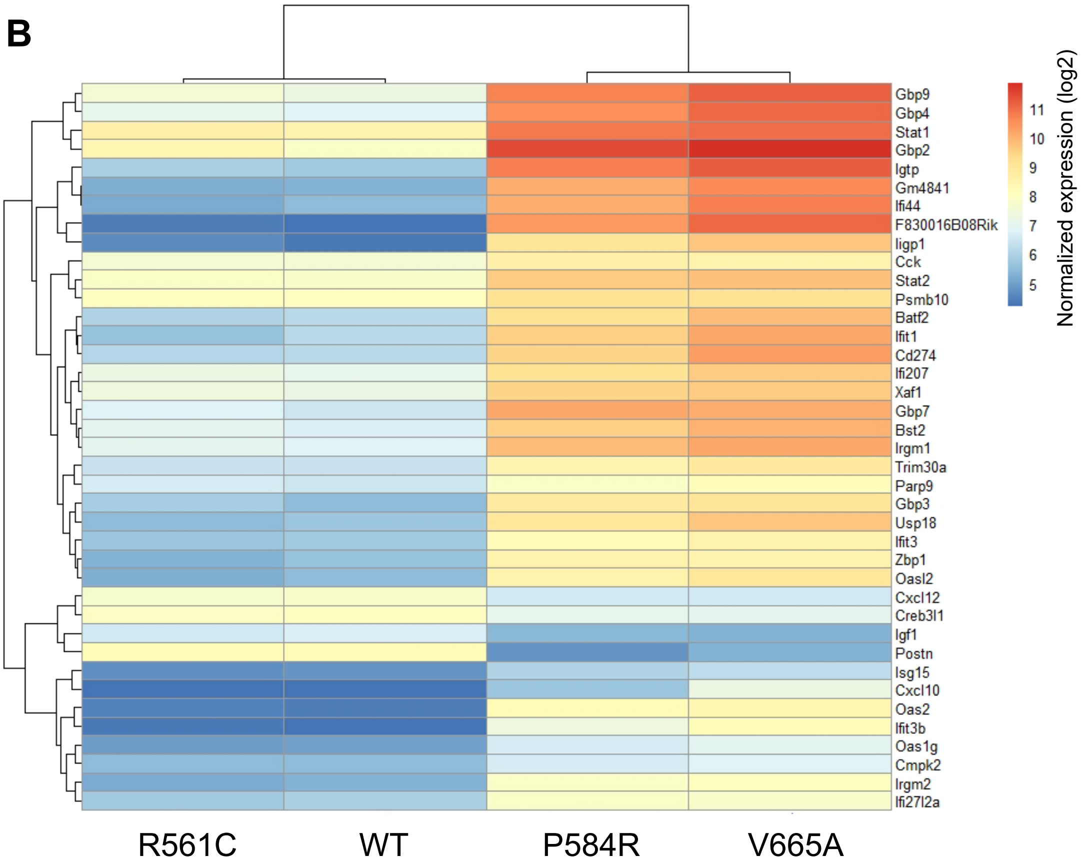

V665A and P584R trigger an interferon-like transcriptional response.

NIH3T3 cells expressing the indicated receptor were produced as described in figure 3. Total RNA was extracted and used to hydridize Affymetrix microarrays (Clariom D array, #520851), according to the manufacturer instructions. (A) Using the Bioconductor limma package, we identified 128 known transcripts that were up-regulated at least two fold in cells expressing βV665A compared to the wild-type receptor. This list was analyzed using DAVID Bioinformatics Resources (https://david.abcc.ncifcrf.gov, version 6.8). The top ten gene ontology terms are shown.
(B) The expression levels of interferon target genes is shown in the four analyzed conditions.
