# Supplementary material for: Penttinen syndrome‐associated PDGFRB Val665Ala variant causes aberrant constitutive STAT1 signalling
Source: J Cell Mol Med. 2022 Jun 10;26(14):3902–12. doi: 10.1111/jcmm.17427 (PMC9279580; doi:10.1111/jcmm.17427)
Supplement: Supplementary file 3 — Figure S3 [file JCMM-26-3902-s002.pdf]

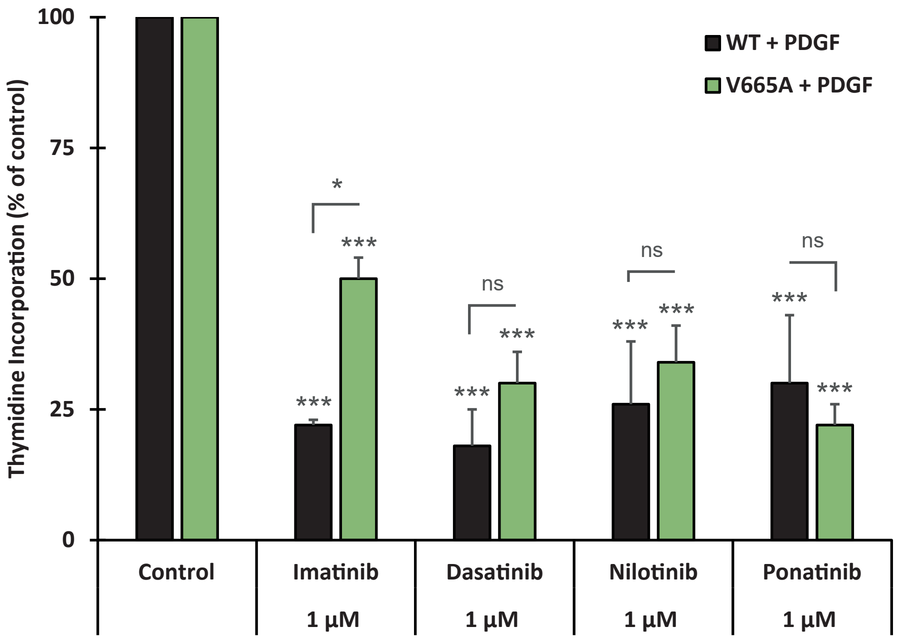

Supplementary Figure S3.

Dasatinib, nilotinib and ponatinib inhibit βV665A activity.

Ba/F3 cells were treated with the indicated TKI (1 µM) for 24 h, as described in Figure 7C, in the presence of PDGF to stimulate cell proliferation. Four hours before harvest, [3H]-thymidine was added to each well. Radiolabeled thymidine incorporation was quantified and compared to untreated cells, used as a reference. The average of three independent experiments is shown with SEM and statistical analysis (Student's *t* test).
