# Supplementary material for: Penttinen syndrome‐associated PDGFRB Val665Ala variant causes aberrant constitutive STAT1 signalling
Source: J Cell Mol Med. 2022 Jun 10;26(14):3902–12. doi: 10.1111/jcmm.17427 (PMC9279580; doi:10.1111/jcmm.17427)
Supplement: Supplementary file 4 — Tables S1‐S4 [file JCMM-26-3902-s003.pdf]

Supplementary Table 1. List of antibodies used in Western Blot.

**Antibody name**	**Company**	**Dilution**
Anti-PY99	Santa Cruz Biotechnology (sc-7020)	1/200
Anti-PDGFRβ (Ct-terminus)	Santa Cruz Biotechnology (sc-432)	1/400
Anti-PDGFRβ (Ct-terminus)	Cell Signaling Technology (3169)	1/1000
Anti-phospho-STAT1 (Tyr701)	Cell Signaling Technology (9167)	1/500
Anti-STAT1	BD Biosciences (610115)	1/1000
Anti-phospho-STAT3 (Tyr705)	Cell Signaling Technology (9131)	1/500
Anti-STAT3	Cell Signaling Technology (9132)	1/1000
Anti-phospho-STAT5 (Tyr694)	Cell Signaling Technology (9351)	1/1000
Anti-STAT5	Santa Cruz Biotechnology (sc-835)	1/1000
Anti-phospho-AKT1 (Ser473)	Cell Signaling Technology (9018)	1/1000
Anti-AKT	Cell Signaling Technology (9272)	1/1000
Anti-phospho-PLCγ1 (Tyr783)	Cell Signaling Technology (2821)	1/1000
Anti-PLCγ1	Cell Signaling Technology (2822)	1/1000
Anti-ACTB	Sigma (A5441)	1/2000
Anti-Calnexin	Enzo (ADI-SPA-860-D)	1/1000
Anti-rabbit IgG	Cell Signaling Technology (7074)	1/5000
Anti-mouse IgG	Cell Signaling Technology (7076)	1/5000

Supplementary Table 2. List of antibodies used in Flow Cytometry.

**Antibody name**	**Company**	**Dilution**
Anti-human PDGFRβ	Homemade (AH17.2, reference [20])	1/300
F'2 Fragment Donkey Anti-Mouse IgG coupled to R-Phycoerythrin	Jackson ImmunoResearch (715-116-150)	1/100

Supplementary Table 3. List of primers used for Real-Time PCR in NIH3T3 cells.

Target	Primers sequence (5'-3')	Tm
PDGFRB	Forward GACCCCAAACCCGAGGTT<br>Reverse ATGGTTGAGGAGGTGTTGACTT	60°C
CXCL9	Forward ACGGAGATCAAACCTGCCTA<br>Reverse TTCCTTGAACGACGACGACT	60°C
CXCL10	Forward CATCCCTGCGAGCCTATCC<br>Reverse CATCTCTGCTCATCATTCTTTTTCA	60°C
CXCL11	Forward ACTATGAAGGCTCATAAACGACAA<br>Reverse TTCTTATTGGAGGGCTCACAG	60°C
IRF1	Forward ACTGTCACCGTGTGTCGTC<br>Reverse TCTGTTGCGGCTTCGGAGG	60°C
USP18	Forward TGCAGGGTCTGTTCACCATC<br>Reverse GCACATGTCGGAGCTTGCTA	60°C
OASL2	Forward GGATGCCTGGGAGAGAATCG<br>Reverse TCGCCTGCTCTTCGAAACTG	60°C
ACTINE B	Forward CTCTGGCTCCTAGCACCATGAAG<br>Reverse GCTGGAAGGTGGACAGTGAG	60°C

Supplementary Table 4. List of primers used for PDGFRB site-directed mutagenesis.

Mutation	Primers sequence (5'-3')	Tm
p.R561C	Forward TTTGGCAGAAGAAGCCATGTTACGAGATCCGATGG<br>Reverse CCATCGGATCTCGTAACATGGCTTCTTCTGCCAAA	55°C
p.W566R	Forward GCCACGTTACGAGATCCGAAGGAAGGTGATTGAG<br>Reverse CTCAATCACCTTCCTTCGGATCTCGTAACGTGGC	55°C
p.P584R	Forward ATCTACGTGGACCGCATGCAGCTGCCC<br>Reverse GGGCAGCTGCATGCGGTCCACGTAGAT	55°C
p.V665A	Forward CCACCTGAACGTGGCCAACCTGTTGGGGG<br>Reverse CCCCCAACAGGTTGGCCACGTTCAGGTGG	55°C
p.N666H	Forward CCTGAACGTGGTCCACCTGTTGGGGGC<br>Reverse GCCCCCAACAGGTGGACCACGTTCAGG	55°C
p.N666K	Forward CCTGAACGTGGTCAAGCTGTTGGGGGC<br>Reverse GCCCCCAACAGCTTGACCACGTTCAGG	55°C
